# Efficacy and safety of CT-P6 versus reference trastuzumab in HER2-positive early breast cancer: updated results of a randomised phase 3 trial

**DOI:** 10.1007/s00280-019-03920-4

**Published:** 2019-08-19

**Authors:** F. J. Esteva, Y. V. Baranau, V. Baryash, A. Manikhas, V. Moiseyenko, G. Dzagnidze, E. Zhavrid, D. Boliukh, D. Stroyakovskiy, J. Pikiel, A. E. Eniu, R. K. Li, A. V. Rusyn, B. Tiangco, S. J. Lee, S. Young Lee, S. Y. Yu, J. Stebbing

**Affiliations:** 1 Perlmutter Cancer Center, NYU Langone Health, 160 E 34th Street, New York, 10016 USA; 2 New York University Langone Medical Center, 550 1st Avenue, New York, 10016 USA; 3 Department of Oncology, Belarusian State Medical University, 220013 Minsk, Belarus; 4 City Clinical Oncology Dispensary, Saint Petersburg, 198255 Russian Federation; 5 GBUZ Saint Petersburg Clinical Research Center of Specialised Types of Care (Oncology), Saint Petersburg, 197758 Russian Federation; 6 S. Khechinashvili University Clinic, Ltd, 0177 Tbilisi, Georgia; 7 N.N. Alexandrov National Cancer Centre of Belarus, 223040 Minsk Region, Belarus; 8 Vinnytsya Regional Clinical Oncology Dispensary, Vinnytsia, 21029 Ukraine; 9 Moscow City Oncology Hospital, Moscow, 143423 Russian Federation; 10 Wojewodzkie Centrum Onkologii, 80-219 Gdańsk, Poland; 11 Cancer Institute “Ion Chiricuta”, 400015 Cluj-Napoca, Romania; 12 St. Luke’s Medical Center, 1102 Quezon City, Philippines; 13 Transcarpathian Regional Clinical Oncology Dispensary, Transcarpathian, 88000 Ukraine; 14 The Medical City, Ortigas Avenue, Pasig City, Philippines; 15 CELLTRION, Inc., Incheon, 22014 Republic of Korea; 16 Division of Cancer, Imperial Centre for Translational and Experimental Medicine, Du Cane Road, London, W12 0HS UK; 17 Imperial College Healthcare NHS Trust, Charing Cross Hospital, Fulham Palace Road, London, W6 8RF UK

**Keywords:** Trastuzumab, Biosimilar, CT-P6, HER2 positive, Adjuvant, Breast cancer

## Abstract

**Purpose:**

Neoadjuvant CT-P6, a trastuzumab biosimilar, demonstrated equivalent efficacy to reference trastuzumab in a phase 3 trial of HER2-positive early-stage breast cancer (EBC) (NCT02162667). We report post hoc analyses evaluating pathological complete response (pCR) and breast pCR alongside additional efficacy and safety measures.

**Methods:**

Following neoadjuvant treatment and surgery, patients received adjuvant CT-P6 or trastuzumab (6 mg/kg) every 3 weeks for ≤ 1 year.

**Results:**

In total, 271 and 278 patients received CT-P6 and trastuzumab, respectively. pCR and breast pCR rates were comparable between treatment groups regardless of age, region, or clinical stage. Overall, 47.6% (CT-P6) and 52.2% (trastuzumab) of patients experienced study drug-related treatment-emergent adverse events (TEAEs), including 17 patients reporting heart failure (CT-P6: 10; trastuzumab: 7). Two CT-P6 and three trastuzumab patients discontinued adjuvant treatment due to TEAEs.

**Conclusion:**

Adjuvant CT-P6 demonstrated comparable efficacy and safety to trastuzumab at 1 year in patients with HER2-positive EBC, supporting CT-P6 and trastuzumab comparability.

**Electronic supplementary material:**

The online version of this article (10.1007/s00280-019-03920-4) contains supplementary material, which is available to authorized users.

## Introduction

Trastuzumab (Herceptin^®^) has altered the management and prognosis of early- and advanced-stage breast cancers that overexpress human epidermal growth factor receptor-2 (HER2). In early breast cancer (EBC) patients, the addition of neoadjuvant trastuzumab to standard chemotherapy significantly improved clinical responses and event-free survival [1, 2], while adjuvant trastuzumab significantly improved long-term disease-free survival (DFS) [3]. Despite clinical benefits, high costs associated with the development of novel biological drugs often translate into high treatment prices [4]. However, the resulting limited access to treatment may be ameliorated by lower priced biosimilars [4]: highly similar versions of approved biological drugs that have undergone extensive comparability testing to demonstrate the absence of clinical differences from their reference product, with regard to efficacy, safety, and purity [5].

CT-P6 (Herzuma^®^), a trastuzumab biosimilar, has equivalent pharmacokinetics and similar safety to the reference product in healthy volunteers [6]. Our phase 3 study compared CT-P6 with trastuzumab in patients with operable HER2-positive EBC, and consisted of a neoadjuvant period involving CT-P6 or trastuzumab treatment with chemotherapy, followed by surgery and subsequent adjuvant treatment. The study met its primary objective of establishing equivalent efficacy of CT-P6 to trastuzumab in patients treated in the neoadjuvant setting [7]. Comparable pharmacokinetics, pharmacodynamics, safety, and immunogenicity were also demonstrated [7]. We report a post hoc subgroup analysis of the primary outcome, additional efficacy outcomes from the adjuvant period and updated overall safety results.

## Materials and methods

### Patients

Patients were female, aged  ≥ 18 years with pathologically confirmed, newly diagnosed, operable, HER2-positive EBC (clinical stage I, II, or IIIA, classified according to the American Joint Committee on Cancer Breast Cancer Staging seventh edition). Full inclusion and exclusion criteria are described in Online Resource 1.

### Study design

This randomised, double-blind, parallel group, active-controlled phase 3 study (ClinicalTrials.gov identifier: NCT02162667) recruited patients from 112 centres in 23 countries [7]. Patients were randomised (1:1) using a computer-generated randomisation schedule and entered the neoadjuvant treatment phase consisting of eight 3-week cycles of CT-P6 (Herzuma^®^; CELLTRION Inc., Incheon, South Korea) or trastuzumab (Herceptin^®^; Genentech, San Francisco, CA, USA) at a loading dose of 8 mg/kg followed by 6 mg/kg for cycles 2–8, with docetaxel and fluorouracil, epirubicin and cyclophosphamide (FEC) as shown in Figure S1 (see Online Resource 3). Following surgery, patients received 6 mg/kg of adjuvant CT-P6 or trastuzumab (per original randomisation [7]), administered as a 90-min intravenous infusion every 3 weeks until ≤ 1 year from the first neoadjuvant dose (excluding the non-treatment period around surgery), or ≤ 10 cycles post-surgery. Patients received radiotherapy and/or hormonal therapy during the adjuvant period at the investigator’s discretion. A post-treatment follow-up period continues for up to 3 years from the date of enrolment of the last patient.

### Assessments and outcome measures

The primary efficacy endpoint, pathological complete response (pCR) was assessed using surgical resection specimens [7]. Breast pCR was defined as the absence of invasive tumour cells in the breast, which included both pCR of breast and axillary nodes regardless of ductal carcinoma in situ and pCR of the breast only. pCR rates were assessed in subgroups defined according to age, region, and clinical disease stage (excluding stage IIIB/IIIC/IV disease due to small sample sizes).

Efficacy endpoints assessed during the adjuvant period included progressive disease (PD), determined by physical examination and mammogram, and the proportions of patients receiving post-surgery radiotherapy or hormonal therapy. DFS and progression-free survival (PFS) will be evaluated in future analyses.

Safety endpoints were assessed throughout the study, or for  ≥ 1 year from the first administration of study drug in patients who discontinued treatment. Endpoints included incidence and severity of treatment-emergent adverse events (TEAEs) according to NCI CTCAE version 4.03, incidence of TEAEs of special interest including infusion-related reactions and cardiotoxicity (mean change from baseline in left ventricular ejection fraction [LVEF]), and immunogenicity (incidence of antidrug antibody).

### Statistical analysis

Sample size was calculated as previously reported [7]. A point estimate and the exact 95% confidence interval (CI) for the difference between treatment groups for the proportion of patients achieving pCR was calculated using the exact unconditional approach. Efficacy analyses were performed in the intent-to-treat (ITT) population and the per-protocol analysis set (PPS). Safety was analysed in the safety analysis set, comprising all patients who received  ≥ 1 (full or partial) dose of study drug.

## Results

### Patients and treatment

**Fig. 1:**
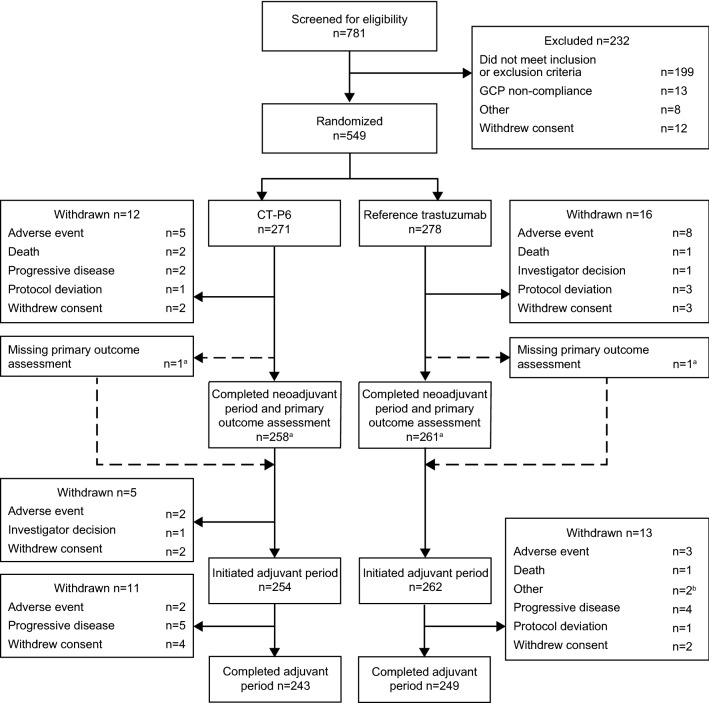
Patient flow diagram. ^a^One patient each from the CT-P6 and trastuzumab treatment groups completed the neoadjuvant period, underwent surgery, and initiated the adjuvant period, but did not complete pCR assessment due to lost pathological samples. ^b^Due to relocation (*n* = 1) and due to being unable to visit treatment site within the visit window (*n* = 1). *GCP* good clinical practice, *pCR* pathological complete response

Of 781 patients screened for enrolment, 549 were randomised to CT-P6 (*n* = 271) and trastuzumab (*n* = 278) and comprised the ITT population (Fig. 1). Most patients completed the neoadjuvant period and pCR assessment [CT-P6: *n* = 258 (95.2%); trastuzumab: *n* = 261 (93.9%)]. Overall, 254 (93.7%) CT-P6 and 262 (94.2%) trastuzumab patients initiated the adjuvant period. Most patients completed the adjuvant period [CT-P6: *n* = 243 (89.7%); trastuzumab: *n* = 249 (89.6%)]. PD was the most frequently reported reason for discontinuation (Fig. 1). Baseline disease characteristics of patients have been presented previously [7]. No notable differences were found between treatment groups (see Online Resource 2, Table S1).

### Efficacy

In the ITT population, pCR rates were comparable between CT-P6 and trastuzumab regardless of age, region, or clinical disease stage (Table 1). The exact 95% CI for the estimated treatment difference in pCR rates demonstrated that results were comparable, with no statistical differences between groups in the subgroups assessed. Similar results were observed for breast pCR rates (Table 1).

**Table 1:** Subgroup analysis of pCR and breast pCR (intent-to-treat population)

Subgroup	CT-P6 (*N* = 271)	Trastuzumab (*N* = 278)	Estimated treatment difference (95% CI)
Age			
pCR			
≥ 65 years	14/31 (45.2%; 27.3–64.0)	20/40 (50.0%; 33.8–66.2)	− 0.05 (− 0.28 to 0.19)
< 65 years	104/240 (43.3%; 37.0–49.9)	111/238 (46.6%; 40.2–53.2)	− 0.03 (− 0.12 to 0.06)
Breast pCR			
≥ 65 years	17/31 (54.8%; 36.0–72.7)	25/40 (62.5%; 45.8–77.3)	− 0.08 (− 0.31 to 0.16)
< 65 years	116/240 (48.3%; 41.9–54.9)	120/238 (50.4%; 43.9–56.9)	− 0.02 (− 0.11 to 0.07)
Region			
pCR			
EMEA	92/209 (44.0%; 37.2–51.0)	107/222 (48.2%; 41.5–55.0)	− 0.04 (− 0.14 to 0.05)
Asia	21/50 (42.0%; 28.2–56.8)	19/46 (41.3%; 27.0–56.8)	0.01 (− 0.19 to 0.21)
America	5/12 (41.7%; 15.2–72.3)	5/10 (50.0%; 18.7–81.3)	− 0.08 (− 0.49 to 0.34)
Breast pCR			
EMEA	103/209 (49.3%; 42.3–56.3)	119/222 (53.6%; 46.8–60.3)	− 0.04 (− 0.14 to 0.05)
Asia	23/50 (46.0%; 31.8–60.7)	21/46 (45.7%; 30.9–61.0)	< 0.01 (− 0.20 to 0.21)
America	7/12 (58.3%; 27.7–84.8)	5/10 (50.0%; 18.7–81.3)	0.08 (− 0.34 to 0.49)
Disease stage^a^			
pCR			
I	13/23 (56.5%; 34.5–76.8)	14/31 (45.2%; 27.3–64.0)	0.11 (− 0.16 to 0.37)
IIA	31/75 (41.3%; 30.1–53.3)	41/86 (47.7%; 36.8–58.7)	− 0.06 (− 0.22 to 0.09)
IIB	52/105 (49.5%; 39.6–59.5)	56/98 (57.1%; 46.7–67.1)	− 0.08 (− 0.21 to 0.06)
IIIA	21/64 (32.8%; 21.6–45.7)	19/61 (31.1%; 19.9–44.3)	0.02 (− 0.16 to 0.19)
Breast pCR			
I	14/23 (60.9%; 38.5–80.3)	14/31 (45.2%; 27.3–64.0)	0.16 (− 0.12 to 0.41)
IIA	35/75 (46.7%; 35.1–58.6)	44/86 (51.2%; 40.1–62.1)	− 0.05 (− 0.20 to 0.11)
IIB	55/105 (52.4%; 42.4–62.2)	62/98 (63.3%; 52.9–72.8)	− 0.11 (− 0.24 to 0.03)
IIIA	28/64 (43.8%; 31.4–56.7)	24/61 (39.3%; 27.1–52.7)	0.04 (− 0.13 to 0.22)

Data are *n*/*N* (%; 95% CI)

*CI* confidence interval, *EMEA* Europe, Middle East, and Africa, *pCR* pathological complete response

^a^pCR rates in patients with stage IIIB, IIIC, and IV subgroups were not included due to small sample sizes

Fifteen patients in the ITT population experienced recurrent or PD at 1 year [CT-P6: *n* = 9 (3.3%); trastuzumab: *n* = 6, (2.2%)]. Results were similar in the PPS: six (2.4%) patients in the CT-P6 group and five (2.0%) patients in the trastuzumab group had PD.

Of the patients who underwent surgery in the ITT population, a similar proportion in both treatment groups was subsequently treated with radiotherapy [CT-P6: *n* = 142 (55.0%); trastuzumab: *n* = 131 (50.2%)]. Post-surgery radiotherapy was most frequently performed on the breast in both treatment groups (Table 2). Results in the PPS were similar (see Online Resource 2, Table S2).

**Table 2:** Summary of post-surgery radiotherapy and hormonal therapy (intent-to-treat population)

	CT-P6 (*N* = 271)	Trastuzumab (*N* = 278)
Patients with surgery, *n* (%)	258 (95.2)	261 (93.9)
Patients with ≥ 1 RT, *n* (%)	142 (55.0)	131 (50.2)
Breast only	60 (23.3)	60 (23.0)
Breast + axilla only	7 (2.7)	15 (5.7)
Breast + SCV/IMC/other ± axilla	57 (22.1)	48 (18.4)
Breast + other ± axilla	13 (5.0)	9 (3.4)
Breast + axilla + SCV ± other	26 (10.1)	20 (7.7)
Breast + axilla + SCV + IMC ± other	3 (1.2)	3 (1.1)
Breast + SCV + IMC ± other	1 (0.4)	2 (0.8)
Other^a^	18 (7.0)	8 (3.1)
Patients with ≥ 1 hormonal therapy, *n* (%)	102 (39.5)	99 (37.9)
Anastrozole	23 (8.9)	20 (7.7)
Exemestane	0	2 (0.8)
Letrozole	17 (6.6)	20 (7.7)
Tamoxifen^b^	63 (24.4)	55 (21.1)
Toremifene	2 (0.8)	1 (0.4)
Goserelin^b^	14 (5.4)	9 (3.4)
Leuprorelin acetate	1 (0.4)	1 (0.4)

The denominator for percentage was the number of patients who had breast surgery during the neoadjuvant period in the ITT population

*IMC* internal mammary chain, *ITT* intent-to-treat, *PPS* per-protocol set, *RT* radiotherapy, *SCV* supraclavicular

^a^All other region combinations not shown in the preceding list

^b^Two patients in the CT-P6 treatment group who initiated hormonal treatment were excluded from the PPS as these were considered to be major protocol deviations

The proportion of hormone receptor-positive patients treated with hormonal therapy was comparable between treatment groups (Table 2). Overall, 201 (38.7%) patients who underwent surgery in the ITT population received  ≥ 1 post-surgery hormonal therapy [CT-P6: 102 (39.5%); trastuzumab: 99 (37.9%) patients]. The most frequent hormonal therapies were tamoxifen, anastrozole, and letrozole. Four patients (receiving trastuzumab) had oophorectomies after the assessment of the primary endpoint.

### Safety

The mean (standard deviation) relative dose intensity (%) of study drug during the neoadjuvant period was similar between treatment groups [CT-P6: 97.5% (2.91); trastuzumab: 97.3% (2.90)]. During the adjuvant period, the relative dose intensity was 98.5% (2.97) and 98.8% (2.27), respectively.

The number (%) of patients experiencing  ≥ 1 TEAE during the 1-year study period was similar between groups [CT-P6: 263 (97.0%); trastuzumab: 265 (95.3%) patients; Table 3]. The number of patients experiencing at least one study drug-related TEAE was 129 (47.6%, CT-P6) and 145 (52.2%, trastuzumab). The most frequent TEAEs considered related to study drug in the CT-P6 group were rash (9.2%), asthenia (8.9%), infusion-related reaction (8.1%), alopecia (7.7%), and neutropenia (7.0%), while these were neutropenia (12.6%), anaemia (9.4%), alopecia (9.0%), asthenia (7.9%), and nausea (7.2%) in the trastuzumab group (Table 3). The number of patients experiencing  ≥ 1 treatment-emergent serious adverse event (SAE) was 20 (7.4%, CT-P6) and 33 (11.9%, trastuzumab) (Table 3). A similar proportion of patients in each group experienced  ≥ 1 study drug-related treatment-emergent SAE. In the CT-P6 group, five (1.8%) patients experienced seven study drug-related SAEs [febrile neutropenia (*n* = 4) and dehydration, neutropenia, and acute pancreatitis (*n* = 1 each)]. In the trastuzumab group, eight (2.9%) patients experienced nine study drug-related SAEs [hypokalaemia and neutropenia (*n* = 2 each) and febrile neutropenia, acute myocardial infarction, congestive cardiomyopathy, anaemia, and cerebral infarction (*n* = 1 each)]. All cases of treatment-related febrile neutropenia occurred in the neoadjuvant period and were due to study drug and docetaxel/FEC treatment. Overall, a similar proportion of patients in each group experienced TEAEs leading to permanent study drug discontinuation [CT-P6: *n* = 11 (4.1%); trastuzumab: *n* = 13 (4.7%)]. There was one study drug-related SAE in each treatment group during the adjuvant period (see Online Resource 2, Table S3). Four TEAEs leading to death occurred during the study (two cases in each treatment group): three during the neoadjuvant period [7] and one during the adjuvant period, due to an aortic dissection that was considered unrelated to study drug (trastuzumab group).

**Table 3:** Summary of adverse events at 1 year^a^ (safety population)

	CT-P6 (*N* = 271)	Trastuzumab (*N* = 278)
Overview of TEAEs		
Total number of TEAEs	2880	3130
Patients experiencing ≥ 1 TEAEs	263 (97.0)	265 (95.3)
Grade 1 or 2	158 (58.3)	153 (55.0)
Grade ≥ 3	105 (38.7)	112 (40.3)
Treatment related^b^	129 (47.6)	145 (52.2)
Total number of treatment-emergent SAEs	26	46
Patients experiencing ≥ 1 treatment-emergent SAEs	20 (7.4)	33 (11.9)
Grade 1 or 2	3 (1.1)	6 (2.2)
Grade ≥ 3	17 (6.3)	27 (9.7)
Treatment related	5 (1.8)	8 (2.9)
TEAEs leading to discontinuation	11 (4.1)	13 (4.7)
TEAEs leading to death	2 (0.7)	2 (0.7)
TEAEs of special interest		
Cardiac disorders	30 (11.1)	37 (13.3)
Treatment related	20 (7.4)	24 (8.6)
Infusion-related reactions	31 (11.4)	29 (10.4)
Treatment related	22 (8.1)	18 (6.5)
Treatment-related TEAEs reported in ≥ 5% of either treatment group		
Alanine aminotransferase increased	4 (1.5)	16 (5.8)
Alopecia	21 (7.7)	25 (9.0)
Anaemia	11 (4.1)	26 (9.4)
Aspartate aminotransferase increased	2 (0.7)	15 (5.4)
Asthenia	24 (8.9)	22 (7.9)
Diarrhoea	14 (5.2)	12 (4.3)
Ejection fraction decreased	19 (7.0)	9 (3.2)
Infusion-related reaction	22 (8.1)	18 (6.5)
Leukopenia	7 (2.6)	18 (6.5)
Nausea	15 (5.5)	20 (7.2)
Neutropenia	19 (7.0)	35 (12.6)
Rash	25 (9.2)	11 (4.0)

Data are *n* or *n* (%). The total number of TEAEs includes all patient events. At each level of summarisation, a patient was counted once if the patient reported one or more events. Only the most severe event is counted

*SAE* serious adverse event, *TEAE* treatment-emergent adverse event

^a^Neoadjuvant period, surgery, and adjuvant period, or at least 1 year (including follow-up) from the first administration of study drug in the neoadjuvant period in patients who discontinued treatment early without completing the adjuvant phase

^b^TEAEs were considered to be related to study drug if the relationship was defined as ‘possible’, ‘probable’, or ‘definite’

**Fig. 2:**
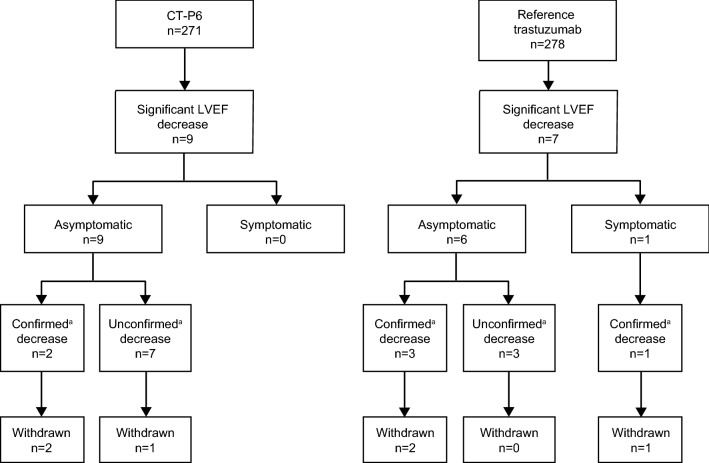
Overall significant decrease in left ventricular ejection fraction. ^a^If LVEF decreased by ten ejection fraction points from baseline and decreased below an absolute value of 50%, LVEF decrease was confirmed by reassessment within 3 weeks to consider treatment discontinuation. *LVEF* left ventricular ejection fraction

TEAEs due to heart failure were reported in 17 patients during the study [CT-P6: *n* = 10 (3.7%); trastuzumab: *n* = 7 (2.5%)] and were mostly considered related to study treatment [CT-P6: *n* = 9 (3.3%); trastuzumab: *n* = 6 (2.2%)]. Of these, 16 patients reported a significant decrease in LVEF [CT-P6: *n* = 9 (3.3%); trastuzumab: *n* = 7 (2.5%)], defined as a decrease of  ≥ 10% from baseline value and below an absolute value of 50%. The remaining patient had no significant LVEF decrease but an asymptomatic drop in LVEF that required treatment. Investigators reported this case as a TEAE due to cardiac failure. Of 16 patients with a significant decrease in LVEF, only one (trastuzumab group) exhibited symptoms of LVEF dysfunction (Fig. 2). Most patients maintained normal LVEF function [CT-P6: *n* = 195 (72.0%); trastuzumab: *n* = 210 (75.5%)]. Median LVEF value was maintained over 60% with no notable differences between the two treatment groups at any time point measured (see Online Resource 2, Table S4). Three patients in each treatment group permanently discontinued study treatment (Fig. 2).

During the adjuvant period, treatment-related infusion-related reactions were reported in 11 (4.1%, CT-P6) and 5 (1.8%, trastuzumab) patients (see Online Resource 2, Table S3). All were grade 1/2 in intensity. There were no notable differences between the two groups in safety assessments. All post-infusion antidrug antibody results were negative throughout the study.

## Discussion

Updated results of this phase 3 study support the biosimilarity of CT-P6 and trastuzumab previously observed during the neoadjuvant phase [7]. Post hoc analysis of the primary efficacy outcome demonstrated comparable pCR rates between treatment groups and across subgroups. CT-P6 was as effective as trastuzumab in preventing PD during the adjuvant period: only a small and comparable number of patients experienced PD in each treatment group. CT-P6 was well tolerated, exhibiting a similar safety profile to trastuzumab.

Benefits of adjuvant trastuzumab treatment have been demonstrated across numerous, varied studies [8]. While it is difficult to compare pCR rates with historical data due to different grouping strategies, in this study, the pCR rate in trastuzumab-treated patients was not affected by region, or by age, in line with some previous observations [9]. A lower pCR rate was observed among patients with later stage disease, per other reports [10, 11]. Nevertheless, pCR rates were similar between trastuzumab and CT-P6 groups, across subgroups.

CT-P6 and trastuzumab demonstrated comparable tolerability up to and including the adjuvant period, and no further study drug-related deaths occurred, supporting the comparability of CT-P6 and trastuzumab safety observed in a phase 1 trial [6]. In the current study, the incidence of TEAEs due to heart failure and median LVEF decrease was comparable between treatment groups, and consistent with previous studies [1, 12]. In the NOAH trial [12], cardiac adverse events were reported in 11% of trastuzumab-treated patients. This compares with 11.1% (CT-P6) and 13.3% (trastuzumab) reported here. The incidence of significant LVEF decreases was low in both treatment groups [3.3% (CT-P6) and 2.5% (trastuzumab)], consistent with the HERA trial (3.0%) [13], demonstrating a low and comparable risk of cardiotoxicity with adjuvant CT-P6 or trastuzumab treatment.

Long-term survival benefits have been demonstrated with adjuvant trastuzumab in EBC [2, 3]. While this approach can be cost-effective, analyses are particularly sensitive to the estimated duration of treatment benefit [14], and evidence suggests that trastuzumab is not cost-effective in low-income countries [15]. Trastuzumab biosimilars can deliver efficacy and safety equivalent to the reference product at a significantly reduced cost, improving cost-effectiveness and access to this beneficial treatment [4].

Limitations of our study include the post hoc nature of the subgroup analyses and the current lack of long-term efficacy data; pCR is only a surrogate for DFS and PFS. A 3-year post-treatment follow-up period is ongoing: future analyses will assess the long-term equivalence of CT-P6 to trastuzumab, although the trial is not powered for survival [7]. The clinical relevance of the data reported herein must be evaluated in this context.

In conclusion, our trial demonstrated that neoadjuvant CT-P6 had comparable efficacy to trastuzumab regardless of patient subgroup analysed. When used as adjuvant therapy following neoadjuvant treatment, CT-P6 demonstrated comparability to trastuzumab in terms of preventing PD. Adjuvant CT-P6 was well tolerated with a similar safety and cardiotoxicity profile to trastuzumab in patients with HER2-positive EBC.

## Electronic supplementary material

Below is the link to the electronic supplementary material.

Supplementary material 1 (PDF 83 kb)

Supplementary material 2 (PDF 125 kb)

Supplementary material 3 (PDF 104 kb)

## Data Availability

All available data are reported in the manuscript and supplementary files. Other additional documents related to the study (for example, protocol, statistical analysis plan, informed consent form) will not be available.

## References

[1] Buzdar AU, Valero V, Ibrahim NK, Francis D, Broglio KR, Theriault RL, Pusztai L, Green MC, Singletary SE, Hunt KK, Sahin AA, Esteva F, Symmans WF, Ewer MS, Buchholz TA, Hortobagyi GN (2007). Neoadjuvant therapy with paclitaxel followed by 5-fluorouracil, epirubicin, and cyclophosphamide chemotherapy and concurrent trastuzumab in human epidermal growth factor receptor 2-positive operable breast cancer: an update of the initial randomized study population and data of additional patients treated with the same regimen. Clin Cancer Res.

[2] Gianni L, Eiermann W, Semiglazov V, Lluch A, Tjulandin S, Zambetti M, Moliterni A, Vazquez F, Byakhov MJ, Lichinitser M, Climent MA, Ciruelos E, Ojeda B, Mansutti M, Bozhok A, Magazzù D, Heinzmann D, Steinseifer J, Valagussa P, Baselga J (2014). Neoadjuvant and adjuvant trastuzumab in patients with HER2-positive locally advanced breast cancer (NOAH): follow-up of a randomised controlled superiority trial with a parallel HER2-negative cohort. Lancet Oncol.

[3] Cameron D, Piccart-Gebhart MJ, Gelber RD, Procter M, Goldhirsch A, de Azambuja E, Castro G, Untch M, Smith I, Gianni L, Baselga J, Al-Sakaff N, Lauer S, McFadden E, Leyland-Jones B, Bell R, Dowsett M, Jackisch C (2017). 11 years’ follow-up of trastuzumab after adjuvant chemotherapy in HER2-positive early breast cancer: final analysis of the HERceptin Adjuvant (HERA) trial. Lancet.

[4] Lammers P, Criscitiello C, Curigliano G, Jacobs I (2014). Barriers to the use of trastuzumab for HER2 + breast cancer and the potential impact of biosimilars: a physician survey in the United States and emerging markets. Pharmaceuticals.

[5] United States Food and Drug Administration (2015) Scientific considerations in demonstrating biosimilarity to a reference product: guidance for industry. https://www.fda.gov/media/82647/download. Accessed 9 Aug 2019

[6] Esteva FJ, Stebbing J, Wood-Horrall RN, Winkle PJ, Lee SY, Lee SJ (2018). A randomised trial comparing the pharmacokinetics and safety of the biosimilar CT-P6 with reference trastuzumab. Cancer Chemother Pharmacol.

[7] Stebbing J, Baranau Y, Baryash V, Manikhas A, Moiseyenko V, Dzagnidze G, Zhavrid E, Boliukh D, Stroyakovskii D, Pikiel J, Eniu A, Komov D, Morar-Bolba G, Li RK, Rusyn A, Lee SJ, Lee SY, Esteva FJ (2017). CT-P6 compared with reference trastuzumab for HER2-positive breast cancer: a randomised, double-blind, active-controlled, phase 3 equivalence trial. Lancet Oncol.

[8] Baselga J, Perez EA, Pienkowski T, Bell R (2006). Adjuvant trastuzumab: a milestone in the treatment of HER-2-positive early breast cancer. Oncologist.

[9] Untch M, Fasching PA, Konecny GE, Hasmuller S, Lebeau A, Kreienberg R, Camara O, Muller V, du Bois A, Kuhn T, Stickeler E, Harbeck N, Hoss C, Kahlert S, Beck T, Fett W, Mehta KM, von Minckwitz G, Loibl S (2011). Pathologic complete response after neoadjuvant chemotherapy plus trastuzumab predicts favorable survival in human epidermal growth factor receptor 2-overexpressing breast cancer: results from the TECHNO trial of the AGO and GBG study groups. J Clin Oncol.

[10] Hamy-Petit A-S, Belin L, Bonsang-Kitzis H, Paquet C, Pierga J-Y, Lerebours F, Cottu P, Rouzier R, Savignoni A, Lae M, Reyal F (2016). Pathological complete response and prognosis after neoadjuvant chemotherapy for HER2-positive breast cancers before and after trastuzumab era: results from a real-life cohort. Br J Cancer.

[11] Buzatto IPC, Ribeiro-Silva A, Andrade JM, Carrara HHA, Silveira WA, Tiezzi DG (2017). Neoadjuvant chemotherapy with trastuzumab in HER2-positive breast cancer: pathologic complete response rate, predictive and prognostic factors. Braz J Med Biol Res.

[12] Gianni L, Eiermann W, Semiglazov V, Manikhas A, Lluch A, Tjulandin S, Zambetti M, Vazquez F, Byakhow M, Lichinitser M, Climent MA, Ciruelos E, Ojeda B, Mansutti M, Bozhok A, Baronio R, Feyereislova A, Barton C, Valagussa P, Baselga J (2010). Neoadjuvant chemotherapy with trastuzumab followed by adjuvant trastuzumab versus neoadjuvant chemotherapy alone, in patients with HER2-positive locally advanced breast cancer (the NOAH trial): a randomised controlled superiority trial with a parallel HER2-negative cohort. Lancet.

[13] Suter TM, Procter M, van Veldhuisen DJ, Muscholl M, Bergh J, Carlomagno C, Perren T, Passalacqua R, Bighin C, Klijn JG, Ageev FT, Hitre E, Groetz J, Iwata H, Knap M, Gnant M, Muehlbauer S, Spence A, Gelber RD, Piccart-Gebhart MJ (2007). Trastuzumab-associated cardiac adverse effects in the herceptin adjuvant trial. J Clin Oncol.

[14] Hall PS, Hulme C, McCabe C, Oluboyede Y, Round J, Cameron DA (2011). Updated cost-effectiveness analysis of trastuzumab for early breast cancer. Pharmacoeconomics.

[15] Aboutorabi A, Hadian M, Ghaderi H, Salehi M, Ghiasipour M (2015). Cost-effectiveness analysis of trastuzumab in the adjuvant treatment for early breast cancer. Glob J Health Sci.

