# Supplementary material for: Efficacy and safety of CT-P6 versus reference trastuzumab in HER2-positive early breast cancer: updated results of a randomised phase 3 trial
Source: Cancer Chemother Pharmacol. 2019 Aug 19;84(4):839–47. doi: 10.1007/s00280-019-03920-4 (PMC6768896; doi:10.1007/s00280-019-03920-4)
Supplement: Supplementary file 1 — Supplementary material 1 (PDF 83 kb) [file 280_2019_3920_MOESM1_ESM.pdf]

Supplementary materials for: Efficacy and safety of CT-P6 versus reference trastuzumab in HER2-positive early breast cancer: updated results of a randomised phase 3 trial

F.J. Esteva, Y.V. Baranau, V. Baryash, A. Manikhas, V. Moiseyenko, G. Dzagnidze, E. Zhavrid, D. Boliukh, D. Stroyakovskiy, J. Pikiel, A.E. Eniu, R.K. Li, A.V. Rusyn, B. Tiangco, S.J. Lee, S. Young Lee, S.Y. Yu and J. Stebbing*

*Corresponding author: Justin Stebbing; Division of Cancer, Imperial Centre for Translational and Experimental Medicine, London, UK; Imperial College Healthcare NHS Trust, Charing Cross Hospital, Fulham Palace Road, London, W6 8RF, UK

Email address: j.stebbing@imperial.ac.uk.

Online Resource 1: Supplementary Methods

Full inclusion and exclusion criteria

Inclusion criteria

Each patient must meet all of the following criteria to be enrolled in this study:

1. Patient is a female 18 years of age or older.
2. Patient who has Eastern Cooperative Oncology Group performance status score of 0 or 1.
3. Patient who has histologically confirmed and newly diagnosed breast cancer.
4. Patient who has clinical stage I, II, or IIIA operable breast adenocarcinoma according to the American Joint Committee on Cancer Breast Cancer Staging 7th edition.

5. At least one measurable lesion by Response Evaluation Criteria in Solid Tumours Version 1.1.

   a. Tumour lesions: ≥10 mm in long axis by computerised tomography (CT) scan

   b. Malignant lymph nodes: ≥15 mm in short axis when assessed by CT scan

6. Patient who has HER2-positive status confirmed locally, defined as 3 + score by immunohistochemistry (IHC). When the IHC result is equivocal (defined as 2+ score), patient who has a positive fluorescence in situ hybridisation or a chromogenic in situ hybridisation result.

7. Patient who has a normal left ventricular ejection fraction (LVEF; ≥55%) at baseline, as determined by either two-dimensional echocardiogram (ECHO) or multiple-gated acquisition (MUGA) scan. If the patient is randomised, the same method of LVEF assessment, ECHO, or MUGA must be used throughout the study.

8. Patient who has known oestrogen receptor and progesterone receptor status.

9. Patient who has adequate bone marrow function, defined as:

   a. Absolute neutrophil count ≥1,500/μL

   b. Haemoglobin ≥10·0 g/dL

   c. Platelets ≥100,000/μL

10. Patient who has adequate hepatic and renal function, defined as:

   a. Aspartate aminotransferase and alanine aminotransferase ≤2·5 × upper limit of normal (ULN)

   b. Total bilirubin ≤1·5 × ULN

   c. Alkaline phosphatase ≤2·5 × ULN

d. Serum creatinine ≤1·5 mg/dL

11. Patient who has the ability to comprehend the full nature and purpose of the study, including possible risks and side effects, to cooperate with the investigator, to understand verbal and/or written instructions, and to comply with the requirements of the entire study.

12. Patient must voluntarily sign an institutional review board/independent ethics committee-approved informed consent form before any study specific procedures.

Exclusion criteria

Patients meeting any of the following criteria will be excluded from the study:

1. Patient who has bilateral breast cancer.
2. Patient who is pregnant or lactating.
3. Patient who has received prior treatment for breast cancer, including chemotherapy, biologic therapy, hormonal therapy, immunotherapy, radiation, or surgery, with the exception of diagnostic biopsy for primary breast cancer.
4. Patient who has received any prior therapy with anthracyclines.
5. Patient who has other concomitant active malignancy or history of malignancy in the past 5 years except treated basal cell carcinoma of the skin or carcinoma in situ of the cervix.
6. Serious cardiac illness or medical conditions that could preclude the use of trastuzumab, specifically: New York Heart Association class ≥2, history of documented congestive heart failure, myocardial infarction, high-risk uncontrolled arrhythmias, angina pectoris requiring medication, clinically significant valvular

disease, evidence of transmural infarction on electrocardiogram, poorly controlled hypertension.

7. Patient who has a current history of infection with hepatitis B, hepatitis C, or infection with human immunodeficiency virus, or who has a positive result to the screening test for those infections.

8. Patient who has had any recent infection requiring a course of systemic anti-infectives that were completed ≤14 days before randomisation (with the exception of uncomplicated urinary tract infection).

9. Patient who is a woman of childbearing potential who is not consenting to use highly effective methods of birth control (e.g., intra-uterine device, barrier methods including condom and diaphragm, also in conjunction with spermicidal jelly, or total abstinence [oral, injectable, or implant hormonal contraceptives are not acceptable]) during treatment and for an additional 7 months after the last administration of the protocol-specified treatment.

10. Patient who is currently receiving treatment with another investigational device or medical product, or for whom less than 30 days or five half-lives ago, whichever is longer, has passed since ending treatment with another investigational device or medical product.

11. Patient who has known sensitivity to any of the products to be administered during the study, including mammalian cell derived drug products, trastuzumab, and murine proteins, or to any of the excipients.

12. Patient who has previously participated in this study.

13. Patient who will likely not be available to complete all protocol-required study visits or procedures.

14. Patient who has history or evidence of any other clinically significant disorder, condition, or disease (with the exception of those outlined above) that, in the opinion of the investigator, would pose a risk to patient safety or interfere with the study evaluation, procedures, or completion.

15. Patient who has pre-existing, clinically significant (>grade 1 by Common Terminology Criteria for Adverse Events v.4.03) peripheral neuropathy.
