# Supplementary material for: Efficacy and safety of CT-P6 versus reference trastuzumab in HER2-positive early breast cancer: updated results of a randomised phase 3 trial
Source: Cancer Chemother Pharmacol. 2019 Aug 19;84(4):839–47. doi: 10.1007/s00280-019-03920-4 (PMC6768896; doi:10.1007/s00280-019-03920-4)
Supplement: Supplementary file 2 — Supplementary material 2 (PDF 125 kb) [file 280_2019_3920_MOESM2_ESM.pdf]

Supplementary materials for: Efficacy and safety of CT-P6 versus reference trastuzumab in HER2-positive early breast cancer: updated results of a randomised phase 3 trial

F.J. Esteva, Y.V. Baranau, V. Baryash, A. Manikhas, V. Moiseyenko, G. Dzagnidze, E. Zhavrid, D. Boliukh, D. Stroyakovskiy, J. Pikiel, A.E. Eniu, R.K. Li, A.V. Rusyn, B. Tiangco, S.J. Lee, S. Young Lee, S.Y. Yu and J. Stebbing*

*Corresponding author: Justin Stebbing; Division of Cancer, Imperial Centre for Translational and Experimental Medicine, London, UK; Imperial College Healthcare NHS Trust, Charing Cross Hospital, Fulham Palace Road, London, W6 8RF, UK

Email address: j.stebbing@imperial.ac.uk.

Online Resource 2: Supplementary Tables

Table S1 Baseline patient characteristics (per-protocol set)

Characteristic	CT-P6 (N=248)	Trastuzumab (N=256)
Age (years)		
Mean (SD)	51.8 (10.86)	51.9 (10.19)
Median (range)	53 (28–78)	52 (24–74)
Race		
Asian	48 (19.4)	44 (17.2)
Black or African American	1 (0.4)	4 (1.6)
Hispanic or Latino	9 (3.6)	8 (3.1)
White	189 (76.2)	198 (77.3)
Other[1]	1 (0.4)	2 (0.8)
Weight (kg)		
Mean (SD)	69.8 (14.93)	71.0 (14.59)
Median (range)	68.0 (44.0–124.0)	69.9 (43.4–120.0)
Region		
EMEA	188 (75.8)	203 (79.3)
America	12 (4.8)	10 (3.9)
Asia	48 (19.4)	43 (16.8)
ECOG performance status (screening)		
0	218 (87.9)	231 (90.2)
1	30 (12.1)	25 (9.8)
Clinical stage[2]		
I	20 (8.1)	30 (11.7)
IIA	69 (27.8)	78 (30.5)
IIB	101 (40.7)	94 (36.7)
IIIA	58 (23.4)	54 (21.1)
Hormone receptor status		
Positive	149 (60.1)	150 (58.6)
Negative	99 (39.9)	106 (41.4)
LVEF		
Median (range)	66.0 (55.2–83.0)	65.7 (55.0–79.0)[3]

[1]Includes one patient in the trastuzumab group for whom race could not be recorded due to local regulations

[2]Patients with stage IIIB, IIIC, IV disease were excluded from the PPS

[3]n=255 (one patient missed LVEF assessment at screening; however, there was no significant LVEF decrease during the neoadjuvant or adjuvant treatment periods)

ECOG=Eastern Cooperative Oncology Group; EMEA=Europe, Middle East, and Africa; LVEF=left ventricular ejection fraction; PPS=per-protocol set; SD=standard deviation

Table S2 Summary of post-surgery radiotherapy and hormonal therapy (per-protocol set)

	**CT-P6 (N=248)**	**Trastuzumab (N=256)**
Patients with surgery, n (%)	248 (100)	256 (100)
Patients with ≥1 RT, n (%)	138 (55.6)	129 (50.4)
Breast only	59 (23.8)	59 (23.0)
Breast + axilla only	7 (2.8)	15 (5.9)
Breast + SCV/IMC/Other ± axilla	54 (21.8)	47 (18.4)
Breast + other ± axilla	13 (5.2)	9 (3.5)
Breast + axilla + SCV ± other	24 (9.7)	19 (7.4)
Breast + axilla + SCV + IMC ± other	3 (1.2)	3 (1.2)
Breast + SCV + IMC ± other	1 (0.4)	2 (0.8)
Other[1]	18 (7.3)	8 (3.1)
Patients with ≥1 hormonal therapy, n (%)	99 (39.9)	97 (37.9)
Anastrozole	23 (9.3)	20 (7.8)
Exemestane	0	2 (0.8)
Letrozole	17 (6.9)	20 (7.8)
Tamoxifen	60 (24.2)	54 (21.1)
Toremifene	2 (0.8)	1 (0.4)
Goserelin	13 (5.2)	8 (3.1)
Leuprorelin acetate	1 (0.4)	1 (0.4)

Note: The denominator for percentage was the number of patients who had breast surgery during the neoadjuvant period in the PPS population

[1]Includes all other region combinations not shown in the preceding list

IMC=internal mammary chain; PPS=per-protocol set; RT=radiotherapy; SCV=supraclavicular

Table S3 Safety data for the adjuvant period only (safety population)

	CT-P6 (N=271)	Trastuzumab (N=278)
Overview of TEAEs		
Total number of TEAEs	450	470
Patients experiencing ≥1 TEAEs	139 (51.3)	147 (52.9)
Grade 1 or 2	130 (48.0)	131 (47.1)
Grade ≥3	9 (3.3)	16 (5.8)
Treatment-related[1]	50 (18.5)	63 (22.7)
Total number of treatment-emergent SAEs	3	14
Patients experiencing ≥1 treatment-emergent SAEs	3 (1.1)	12 (4.3)
Grade 1 or 2	1 (0.4)	3 (1.1)
Grade ≥3	2 (0.7)	9 (3.2)
Treatment-related	1 (0.4)	1 (0.4)
TEAEs leading to discontinuation	2 (0.7)	3 (1.1)
TEAEs leading to death	0	1 (0.4)
TEAEs of special interest		
Cardiac disorders	10 (3.7)	13 (4.7)
Treatment-related	5 (1.8)	8 (2.9)
Infusion-related reactions	11 (4.1)	5 (1.8)
Treatment-related	11 (4.1)	5 (1.8)
Treatment-related TEAEs reported in ≥3% of either treatment group		
Alanine aminotransferase increased	0	9 (3.2)
Anaemia	7 (2.6)	11 (4.0)
Aspartate aminotransferase increased	0	9 (3.2)
Asthenia	8 (3.0)	5 (1.8)
Ejection fraction decreased	11 (4.1)	4 (1.4)
Infusion-related reaction	11 (4.1)	5 (1.8)
Neutropenia	4 (1.5)	10 (3.6)

Note: Data are n or n (%). The total number of TEAEs count included all patient events. At each level of summarisation, a patient was counted once if the patient reported one or more events. Only the most severe event is counted

[1]TEAEs were considered to be related to study drug if the relationship was defined as ‘possible’, ‘probable’ or ‘definite’

SAE=severe adverse event; TEAE=treatment-emergent adverse event

Table S4 Summary of left ventricular ejection fraction measurements up to the end of the adjuvant period[1] (safety population)

	**CT-P6 (N=271)**	**Trastuzumab (N=278)**
LVEF at baseline		
n	271	277
Median (range)	66.0 (55.0–83.0)	66.0 (55.0–79.0)
LVEF after neoadjuvant period cycle 4		
n	262	263
Median (range)	65.0 (38.0–80.0)	65.0 (42.0–85.0)
LVEF at end of neoadjuvant period		
n	266	268
Median (range)	64.0 (44.0–82.0)	64.0 (44.0–78.0)
LVEF before adjuvant period cycle 1		
n	225	226
Median (range)	64.0 (52.0–80.0)	64.0 (51.9–85.0)
LVEF after adjuvant period cycle 3		
n	229	235
Median (range)	63.0 (49.0–85.0)	63.1 (43.1–84.0)
LVEF after adjuvant period cycle 6		
n	235	237
Median (range)	63.0 (43.0–77.0)	64.0 (44.0–79.0)
LVEF at end of adjuvant period		
n	244	258
Median (range)	63.0 (44.0–78.2)	64.0 (33.0–78.0)
Overall worst LVEF value[2]		
n	267	272
Median (range)	60.0 (38.0–70.0)	60.0 (30.0–76.0)
LVEF <50 and decrease of ≥10 from baseline, n (%)	9 (3.3)	7 (2.5)

[1]Includes neoadjuvant period, surgery, and adjuvant period or at least one year (including follow-up) from the first administration of study drug in the neoadjuvant period in patients who discontinued treatment early without completing the adjuvant phase

[2]The overall worst LVEF value for a patient was the lowest post-baseline LVEF value

LVEF=left ventricular ejection fraction
