# Supplementary material for: Efficacy and safety of CT-P6 versus reference trastuzumab in HER2-positive early breast cancer: updated results of a randomised phase 3 trial
Source: Cancer Chemother Pharmacol. 2019 Aug 19;84(4):839–47. doi: 10.1007/s00280-019-03920-4 (PMC6768896; doi:10.1007/s00280-019-03920-4)
Supplement: Supplementary file 3 — Supplementary material 3 (PDF 104 kb) [file 280_2019_3920_MOESM3_ESM.pdf]

Supplementary materials for: Efficacy and safety of CT-P6 versus reference trastuzumab in HER2-positive early breast cancer: updated results of a randomised phase 3 trial

F.J. Esteva, Y.V. Baranau, V. Baryash, A. Manikhas, V. Moiseyenko, G. Dzagnidze, E. Zhavrid, D. Boliukh, D. Stroyakovskiy, J. Pikiel, A.E. Eniu, R.K. Li, A.V. Rusyn, B. Tiangco, S.J. Lee, S. Young Lee, S.Y. Yu and J. Stebbing*

*Corresponding author: Justin Stebbing; Division of Cancer, Imperial Centre for Translational and Experimental Medicine, London, UK; Imperial College Healthcare NHS Trust, Charing Cross Hospital, Fulham Palace Road, London, W6 8RF, UK

Email address: j.stebbing@imperial.ac.uk.

Online Resource 1: Supplementary Figure

Fig. S1 Study design diagram

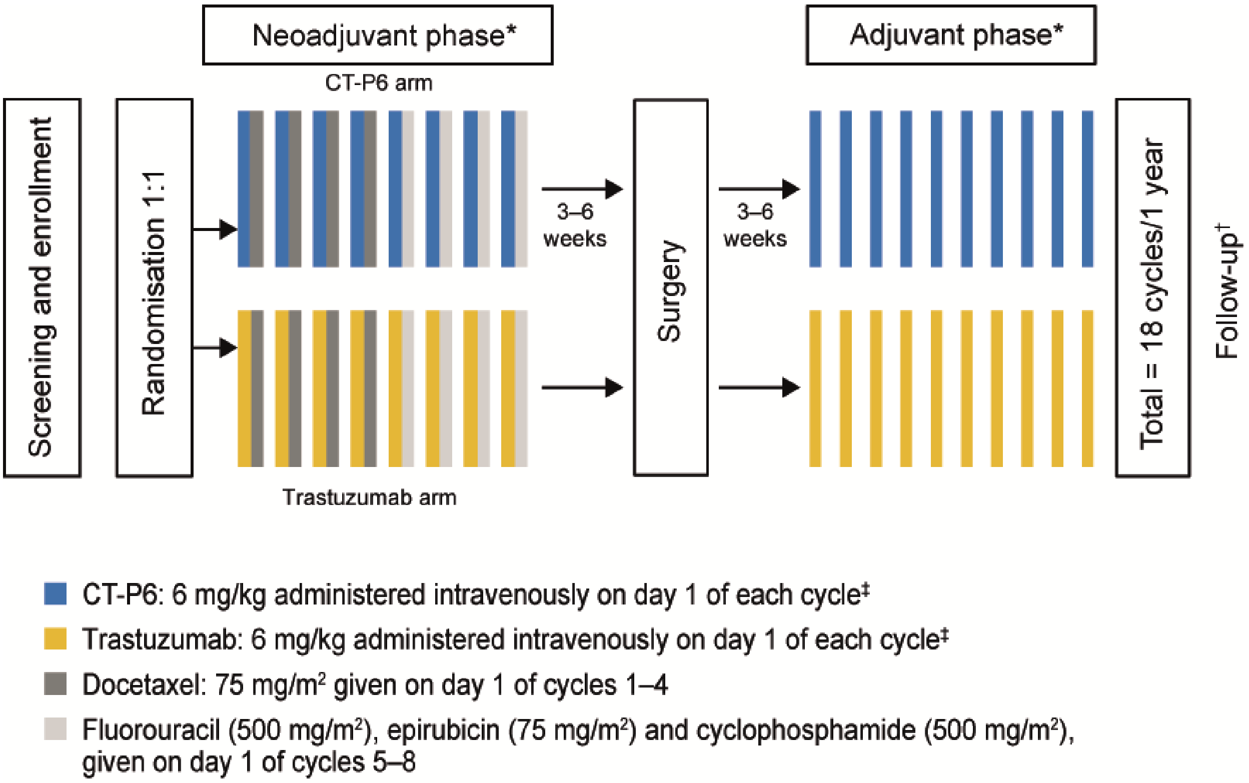

*Each cycle lasted 3 weeks

†Up to 3 years from the date of enrolment of the last patient

‡In place of the usual 6 mg/kg dose, an 8 mg/kg loading dose was administered on day 1 of cycle 1 in the neoadjuvant period only
